# Identifying key outcomes for social prescribing research in the US: a Delphi method study

**DOI:** 10.3389/fpubh.2026.1761684

**Published:** 2026-08-20

**Authors:** Jill Sonke, Nicole Morgan, Daisy Fancourt

**Affiliations:** 1 Center for Arts in Medicine, University of Florida, Gainesville, FL, United States; 2 Biobehavioral Research Group, University College London, London, United Kingdom

**Keywords:** arts prescribing, outcomes, public health, social prescribing, social prescribing outcomes

## Abstract

The US has a history of using social prescribing (SP) to address social determinants of health. Recently, and in alignment with global trends, more SP programs are being developed with a broader focus on community-based social supports and social connection. As SP programs proliferate in the US in the absence of a synthesizable evidence base, this study sought to identify key common outcomes to help enable evidence synthesis to guide practice, policy, research, and investment in social prescribing in the US. This study used a three-phase modified Delphi Method approach to develop a set of key common outcomes for SP research in the US. A mapping review of global social prescribing literature identifying 347 unique outcomes organized in 18 categories of data was followed by two rounds of Delphi Method processes including iterative surveys, group dialogue, and member checking with purposive samples of 17 international and 21 US SP experts and stakeholders. Quantitative and qualitative data were analyzed to create ranked lists of priority patient and system outcomes. The study identified 10 patient outcomes and six system outcomes as priority for study of SP programs in the US. Wellbeing, social connection, and coping/resilience were the three top priority patient outcomes. Health equity, return on investment, and health system cost savings were the top three priority health system outcomes. Consistent study of these key common outcomes has the potential to enable evidence synthesis of social prescribing research and, in turn, drive evidence-based practice, investment, and policy in this fast-growing arena of public health practice in the US.

## 1 Introduction

The US has a decades-long history of working to address social determinants of health amongst patients presenting to healthcare professionals by connecting these patients to resources that help them meet their basic needs, such as food, housing, and transportation (1, 2). These programs, which have operated mostly in primary care settings, include screening for social risks and needs, followed by referral to community social services (2, 3). More recently, the Centers for Medicaid and Medicare Services (CMS) have implemented structures, such as its Accountable Health Communities Model, in which social needs are identified and addressed through community referral and navigation services (4). In 2024, as part of a plan to advance health equity, CMS began requiring in-patient care providers to screen for social determinants (5). Many outpatient services do the same and make community referrals that address social risks and needs.

Increasingly over the past decade, these types of programs have been described as “social prescribing” (SP) and have emerged globally to include a broader focus on social supports, social connection as well as social determinants of health. A recent international study defined SP as “a means for trusted individuals in clinical and community settings to identify that a person has non-medical, health-related social needs and to subsequently connect them to non-clinical supports and services within the community by co-producing a social prescription—a non-medical prescription, to improve health and wellbeing and to strengthen community connections.” (6) Operationalizing this definition for practice, the UK’s National Academy of Social Prescribing defines five categories of SP activities, including arts and culture, heritage, natural environment, physical activity, and advice and information (including referrals to address basic needs, as in the historical US context) (7). SP is now established in at least 40 countries including the US (8), with some focusing on the whole umbrella of SP, and others focusing on sub-sets like arts prescribing and nature/green prescribing (9–11). As an example, in the United Kingdom (UK), SP has become embedded in the national healthcare system, with National Health Service (NHS) support of over 3,500 link workers and with over 3 million people over the past decade (12).

While the global evidence for SP is inconsistent, numerous studies provide promising evidence that it may offer an accessible means to improving wellbeing and mental health outcomes, as it promotes social belonging and inclusion, connectedness, and mental wellbeing (13–16). It has also been shown to decrease the need for and utilization of clinical and mental health services by reducing anxiety and improving physical and mental health (17–20). However, evidence synthesis is limited and reviews to date note the need for common measures and reporting to enable evidence synthesis (16, 21). Given the very different social/political contexts and health systems of the US and UK, where most SP research has been done, specific priority outcomes are needed for measurement of SP in the US context.

Evidence synthesis is critical to guiding evidence-based practice, investment, and policy related to promising health practices such as SP. Core Outcomes Sets (COS) are especially instrumental, as they define recommended priority outcomes for study related to a particular health issue or intervention so results of studies can be more reliably synthesized and compared (22). The aim of this study was to develop a set of key common outcomes (KCOs) as a step toward development of a COS for SP in the US, which in turn can inform future evidence-based SP practice, policy, and investment in the US.

## 2 Materials and methods

This study used a three-phase modified Delphi Method approach to develop a set of key common outcomes for SP research in the US. The Delphi Method is a way of gaining consensus through iterative rounds of questioning and reflection with “experts” or people with experience on a topic (23, 24). As no specific guidelines exist for the development of a key common outcomes set, this study used standards for the development of core outcomes sets as guidance (25).

**Figure 1:**
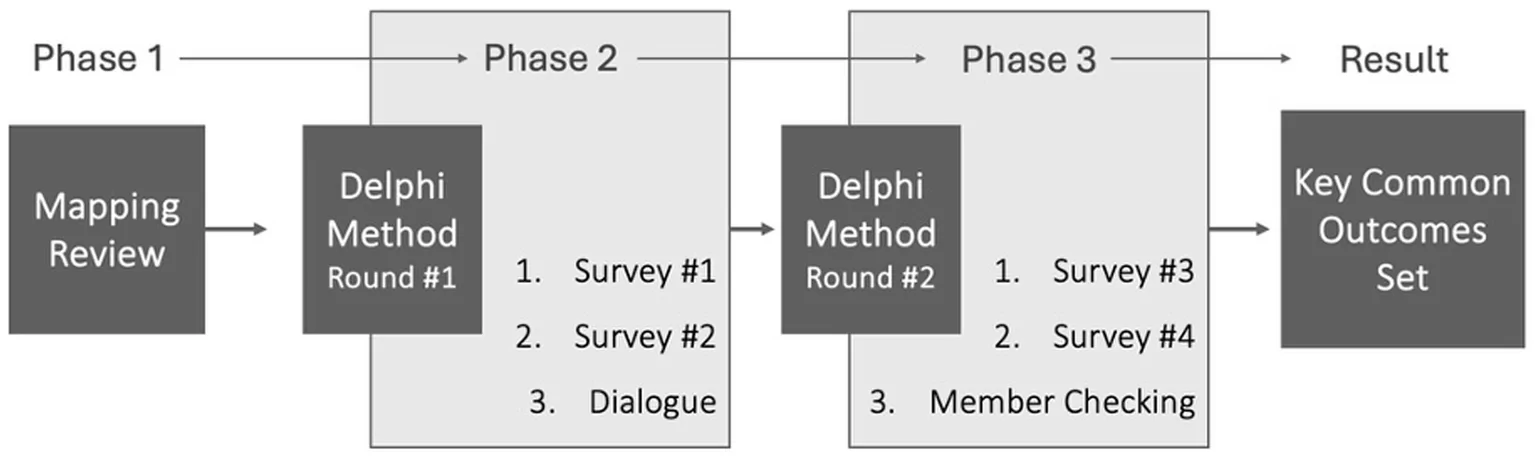
Study design and workflow model showing the three-phase Delphi process leading to the key common outcomes set.

The first phase of the study, conducted between November 2022 and June 2023, was a mapping review of the literature to identify outcomes that have most commonly been studied in 13 countries where SP activities had been documented (26, 27). This work built on and extended the literature review of SP outcomes conducted by Polley et al. (28, 29) in the UK and was published in 2023 (26). The second phase of the study (September 13, 2023) was a Delphi Method process that engaged global experts on SP in two iterative surveys, followed by a group dialogue. The third phase was a Delphi Methods process that engaged SP experts and stakeholders from the US in two iterative surveys, followed by individual member checking. See Figure 1 below.

### 2.1 Mapping review of social prescribing outcomes

A mapping review of existing literature was conducted to identify outcomes that have most commonly been studied as a foundational step in the development of the KCO set [See Sonke et al., (26)]. The review identified 87 studies from the 13 countries cited in the World Health Organization’s (WHO) Social Prescribing Toolkit (27). The review and its search strategy built on a previous study of SP outcomes in the UK (28). With the permission of its authors, the strategy was adapted by a health sciences librarian for this review using a Population, Concept, Context (PCC) conceptual framework. After testing, a final search strategy was created and translated into eight databases. Handsearching gathered grey literature and included snowballing of the systematic and other reviews captured in the database searches. Screening utilized criteria based on the PCC conceptual framework, with nine researchers screening article titles and abstracts, followed by a full-text screening of remaining articles and conflict resolution. Resulting outcomes were extracted from articles that met criteria, including author information, study descriptors such as location, population and sample size, and all outcomes reported as measures or findings. Following extraction, outcomes were verified and categorized into patient outcomes and system outcomes. A second categorization phase developed sub-categories using an inductive qualitative content analysis approach, yielding 10 categories of data. See Sonke et al. (26) for a full report of the mapping review methods, including full inclusion/exclusion criteria, screening process, and PRISMA flow diagram.

### 2.2 Delphi method process round #1

#### 2.2.1 Study participants

Two purposive samples of heterogenous experts and stakeholders in SP were identified by the research team. The first sample of international experts was derived from a list of individuals who had authored key research identified in the mapping review along with individuals who were known by the research team to be established leaders in SP.

#### 2.2.2 Data collection and analysis

The first group of potential international participants were invited via e-mail to participate in a 2-hour Zoom session to complete two iterative surveys, plus group discussion. The invitation was sent to 34 individuals, 21 of whom consented to participate. The participants were provided with a draft manuscript of the mapping review study in advance of the Zoom session, which included the identified and quantified outcomes. The session began with introductions, an overview of the study and review of the mapping review results. After an opportunity to ask questions and to opt out of the study, participants were invited to complete the first outcomes ranking survey (survey #1) via Qualtrics (See surveys in Supplemental materials). Participants completed the survey independently with audio and video muted, only turning cameras on when finished. A 10-min break was provided while the research team created lists of ranked outcomes. Outcomes that were included in the top 10 priority lists of 4 or more people were included in the ranked list for the next round. This list was not meant to represent consensus, but rather to identify highest priority outcomes of interest and eliminate outliers and outcomes of lesser interest for the next stage of consideration. Inclusion of an outcome by 20% of participants or more was deemed to indicate outcomes of reasonable interest. Ranked outcomes lists were then presented, and the group was asked to complete a second survey (survey #2) which gave them the opportunity to change their rankings based on the results of the first survey. After another 10-min break and the same analysis process, the research team presented the second survey results and invited group discussion guided by three questions: (1) “Why did you choose the outcomes you chose?”; (2) “Are there any different or dissenting perspectives that we should consider?”; and (3) “Are there any outcomes that have not but should be considered?.” Discussion was recorded and transcribed. Following the meeting, thematic analysis was conducted by the research team.

### 2.3 Delphi method process round #2

#### 2.3.1 Study participants

The second group of invited participants included individuals from the US who participated in the first Delphi Method process, along with other stakeholders representing various roles in US SP programs, including program organizers, referrers, facilitators, and participants. These individuals were identified through a separate SP study being conducted by the research team.

#### 2.3.2 Data collection and analysis

The second round of surveying (Surveys #3 and #4) was conducted asynchronously with 37 individuals from the US. In the first stage of Round #2, consented participants were asked to review the lists of patient and system outcomes developed during Round #1. They were then invited to respond anonymously with suggestions for changes, deletions, or additions to the priority ranking and comments about their choices. These anonymous responses were collated, and the revised sets of outcomes were sent back to all invitees for final consideration in Survey #4. Participants again had the opportunity to suggest changes, deletions, or additions. The research team collated these responses and conducted a thematic analysis of the comments. Finally, a member checking process invited participants to affirm the final lists of outcomes.

## 3 Results

### 3.1 Mapping review of social prescribing outcomes

A total of 347 unique outcomes were identified from 87 articles. These outcomes were organized across two main categories of patient and system outcomes, with sub-categories that collectively presented a comprehensive view of the outcomes studied. The review identified a total of 278 unique patient outcomes, with the highest prevalence in the *Mental Health* category, followed by *Lifestyle and Behavior*, *Patient/Service User Experience*, *Relationships and Social Connections*, *Physical Health*, *Community Engagement and Belonging*, *Wellbeing*, and *Social Determinants of Health*. The review identified 69 unique system outcomes, with the highest prevalence in the *Healthcare and Service Utilization* category, followed by *Financial and Economic Outcomes*, *Workforce*, *Medication Use and Prescribing*, and *General System Outcomes*. See Sonke et al. (26) for a full report of the mapping review results.

### 3.2 Delphi method processes

#### 3.2.1 Study participants

Participants in this study reported being involved in SP for between 1 and 20 years, with a mean involvement of 4.8 years (SD = 3.9). In Round #1, 17 individuals from four countries (the US, Canada, the UK, and Australia) participated in the Delphi Method process (Surveys #1 and #2). In Round #2, participation varied between the surveys (Surveys #3 and #4). Of the 37 individuals from the US invited to participate, 21 completed Survey #3 and 10 completed Survey #4. Because these surveys were anonymous, the number of individuals who participated in both rounds is unknown. Additional demographics, including disciplines and roles represented, are presented in Table 1 below.

**Table 1:** Delphi method process demographics.

Personal identifiers	Round 1 (*n* = 17) Survey #1 & Survey #2	Round 2 (*n* = 21) Survey #3	Round 2 (*n* = 10) Survey #4
Primary discipline			
Healthcare	24%	33%	20%
Public health	35%	29%	30%
Arts and culture	18%	24%	20%
Other	24%^1^	14%^4^	30%^7^
Primary role related to SP			
SP manager or administrator	12%	29%	30%
SP program/service provider	6%	10%	0%
SP researcher	53%	24%	30%
SP link worker, community health worker or other program liaison	0%	5%	0%
Other	29%^2^	33%^5^	30%^8^
Two or more roles	N/A	N/A	10%^9^
Race/ethnicity			
Asian or Asian American	12%	5%	0%
Black or African American	0%	10%	10%
Latino/a/x or Hispanic origin, such as Mexican, Puerto Rican, or Cuban	6%	10%	20%
Native American or Native Alaskan	0%	0%	0%
Native Hawaiian or Pacific Islander	0%	5%	0%
White or Caucasian	71%	52%	50%
Two or more races/ethnicities	6%^3^	5%^6^	0%
Other	6%	0%	10%^10^
Prefer not to answer	0%	10%	10%
No Response	N/A	5%	N/A
Gender Identity			
Gender non-conforming	6%	5%	0%
Man (cisgender or transgender)	35%	33%	30%
Non-binary	0%	0%	0%
Woman (cisgender or transgender)	53%	52%	60%
Identity not listed	0%	0%	0%
Prefer not to answer	6%	5%	10%
No Response	N/A	5%	N/A

1 Planetary health (*n* = 1), Research (*n* = 1), and Social services/impact (*n* = 2).

2 Global/national advocate (*n* = 1), program developer (*n* = 1), funder (*n* = 1), and public health administrator (*n* = 1), No response (*n* = 1).

3 Asian or Asian American annd White or Caucasian (*n* = 1).

4 Non-profit (*n* = 1), research (*n* = 1), and finance (*n* = 1).

5 Clinicians (*n* = 2), SP field builders (*n* = 2), health department administrator (*n* = 1), and program partners without identified roles (*n* = 2).

6 Asian or Asian American and White or Caucasian (*n* = 1).

7 Research (*n* = 1), finance (*n* = 1), and “arts, culture and health” (*n* = 1).

8 Advocate (*n* = 1), policy professional (*n* = 1) stakeholder (*n* = 1).

9 Program/service provider and researcher (*n* = 1).

10 Caribbean American (*n* = 1).

Finally, seven people from the US participated in member checking. Three were from public health, one from healthcare, two from the arts, and one was a program participant. Demographic information was not collected in this round.

#### 3.2.2 Ranked outcomes

Through its iterative rounds of input, the Delphi process yielded two sets of outcomes—one set of patient outcomes and one set of system outcomes—ranked by priority. Table 2 below shows how the lists of outcomes evolved from the mapping review through the Delphi Method rounds and member checking processes to result in a final list of key common outcomes for SP research in the US. Only two small changes were made as a result of the member checking process. First, loneliness was prioritized over health self-efficacy; and secondly, recognizing more common terminology in the US, “GP visits” was changed to “primary care provider visits.”

**Table 2:** Ranked outcomes from each study component.

Mapping review (global perspective)	Delphi method Round #1 (global perspective)	Delphi Method Round #2 (US perspective)	Final key common outcomes
Patient outcomes – ranked by priority			
1. Mental health	1. Wellbeing	1. Wellbeing	1. Wellbeing
2. Lifestyle and behavior	2. Condition management	2. Social connection	2. Social connection
3. Patient/service user experience	3. Health self-efficacy	3. Coping and resilience	3. Coping and resilience
4. Relationships and social connection	4. Social connection	4. SDoH	4. SDoH
5. Physical health	5. Coping and resilience	5. Loneliness	5. Loneliness
6. Community engagement and belonging	6. Belonging	6. Health self-efficacy	6. Health self-efficacy
7. Wellbeing	7. Loneliness	7. Belonging	7. Belonging
8. Social determinants of health (SDoH)	8. Physical activity	8. Condition management	8. Condition management
	9. SDoH	9. User experience	9. User experience
	10. User experience	10. Physical activity	10. Physical activity
System outcomes – ranked by priority			
1. Healthcare/service utilization	1. Return on Investment	1. Health equity	1. Health equity
2. Financial/economic	2. Health equity	2. Return on Investment	2. Return on Investment
3. Medication use/prescribing	3. Health system cost savings	3. Health system cost savings	3. Health system cost savings
4. General system outcomes	4. System utilization	4. System utilization	4. System utilization
5. Healthcare/service utilization	5. GP visits	5. Workforce outcomes	5. Workforce outcomes
6. Financial/economic	6. Workforce outcomes	6. Primary Care Provider visits	6. Primary Care Provider visits

#### 3.2.3 Qualitative themes

Themes developed from the dialogue in the first round of the Delphi Method process and from member checking were compared to the survey results and used to verify the quantitative rankings (and to settle one tie in rankings). Priorities for outcomes noted in the dialogic and member checking processes were also used to verify the quantitative results. This assessment supported the high priorities for wellbeing, health equity, and return on investment. Five themes emerged from the dialogue and member checking:

##### 3.2.3.1 It is important to allow for breadth in social prescribing outcomes measurement

Participants agreed that there are a broad range of priorities in SP programs, resulting in a broad and inconsistent range of outcomes measurement and reporting. Members of the group emphasized the importance of breadth in health outcomes measurement that can allow for exploration of unique program priorities, in balance with consistency that can enable evidence synthesis. One UK researcher noted that earlier UK core outcomes work was not successful due to the broad range of priorities in SP programs across the NHS.

##### 3.2.3.2 Relationships between outcomes are important

It was noted that multiple outcomes occur concurrently in SP programs, and that the order in which outcomes occur may be important to study. For example, it was recognized that some outcomes may occur before or influence others for service users, such as experiencing hope, connection, or confidence, and other social determinants or drivers of health. There was group agreement that these outcomes should be studied before or alongside health outcomes to understand their relationships.

##### 3.2.3.3 Social prescribing research should focus on wellbeing

There was agreement among participants that SP often seeks to address more general aspects of health and wellbeing, rather than—or alongside—specific clinical outcomes. The group emphasized the importance of patient voices and qualitative data in this regard.

##### 3.2.3.4 Outcomes measurement should be practical and feasible

The group recognized differences between what is easy to measure and what is important to measure. There was agreement that feasibility and practicality of measurement should be a consideration, and that measurement should be undertaken across stakeholder groups. Specifically, stakeholders should be brought together to coordinate who can study what outcomes among the priorities.

##### 3.2.3.5 There is a need for consistency in financial and systems outcome measurement

The group recognized the need and opportunity for more standardized measures and instruments for assessing financial and systems-level outcomes, in addition to the need for clarifying the kinds of financial measures that are important in both the health and social domains.

## 4 Discussion

The results of this modified Delphi Method study offer 10 key common patient outcomes and six key common system outcomes that SP experts and stakeholders in the US agree should be measured in SP research in the US. Such common measurement builds on existing evidence and will help to strengthen the evidence base by making findings more synthesizable.

The methods undertaken to develop this KCO set were very similar to those commonly undertaken to develop a COS. Development of core outcomes for SP in the US was thought by the research team to be premature for several reasons. Firstly, while a broad international definition for SP has recently been established (6), there are variations in how SP is conceptualized and practiced in the US. Secondly, given that evidence related to SP in the US is nascent, there are no long-term or clear patterns in outcomes to serve as a basis for COS development. Additionally, given that SP is not a single intervention, but rather a mechanism for linking people to interventions, sufficient nuance will be needed to address heterogeneity in populations, interventions, and desired outcomes. In the future when systemic review of US SP evidence is possible, development of a COS should be explored. Such a resource should be designed with sufficient nuance and adaptability in acknowledgement of this heterogeneity.

The results of this study align with those of two recent studies, including an umbrella review of SP outcomes and measures that synthesized outcomes and measures from 33 studies, 88% of which were from the UK, finding that more than half measured dimensions of wellbeing, and a prevalence of studies of user experience, mental health, social functioning, GP visits, and return on investment (30). The results also align with Polley et al’s (29) review that identified 67 SP outcomes and organized them into six categories—general, physical, psychological, welfare, spiritual, and social. Like in this study’s mapping review (26), wellbeing was the most commonly studied outcome in the general category. Outcomes related to physical activity, along with risk factors for non-communicable diseases, were commonly noted in the physical health category, and loneliness and social isolation were also commonly studied.

Notably, there were some significant differences between the outcomes identified in the mapping review and those identified as priorities in the Delphi Method process. Most significantly, while mental health was the largest outcomes category in the review, it did not emerge as a single priority in the Delphi Method process. Coping and resilience from that category was a priority in the final list, and other outcomes such as loneliness and belonging are generally linked to mental health. It is possible that respondents considered wellbeing to be a broader category than mental health, and therefore a priority over specific psychopathological symptomatology.

### 4.1 Implications for practice

Alongside resources such as the recent *Arts on Prescription: A Field Guide for US Communities* publication (10), this KCO set serves as a tool that can help SP practitioners, researchers, funders, and policy-makers design, implement, and evaluate SP programs that pursue a broad range of outcomes while contributing to a field-wide dialogue about how to develop interventions that effectively meet the needs of diverse individuals and communities. This KCO set can support development of shared language around roles of SP in the US, which in turn, may enhance understanding for prospective participants and collaborators. Additionally, this KCO set offers opportunities for more routine evaluation of SP in the US, as well as future development of common measures, a minimum dataset, or core outcomes set.

This work also contributes to a care paradigm that seeks to attend to the needs of individuals holistically and supports the growing focus of US health systems on activating community-based social supports for wellness and prevention. The identification of priority system outcomes may contribute to the better integration of healthcare, social care, and community services, which may in turn contribute to health equity and more equitable access to wellness and prevention services. SP presents opportunities for health systems, and their component parts, to make progress on high-level goals related to health equity by expanding care mechanisms upstream to address social and systemic drivers of health through cross-sector partnership and systems alignment (27, 31).

As SP presents opportunities for the US to address critical issues such as loneliness, a more synthesizable evidence base that can guide evidence-based practice, policy and investment is critical. Notably, the previous US Surgeon General launched a major national framework to address loneliness, noting that half of Americans experience loneliness—which poses an even greater health risk than inactivity or obesity—and calling for pro-connection public policies (32–34). This KCO set may help to advance this framework’s goals by contributing to a more robust and synthesizable research agenda for SP related to loneliness.

### 4.2 Key considerations

As was emphasized by study participants, SP programs should be designed to address the unique and specific needs of specific communities. This KCO set is not intended to limit what outcomes are studied, but rather to encourage common study of key outcomes that may be relevant across many programs and that can advance the quality of research as well as enable evidence synthesis, which is critical to the advancement of new and promising health practices.

This study identified as outcomes some concepts that may be better considered groups or categories of outcomes. Most notably, while wellbeing is sometimes measured as a single construct (e.g., using the WHO5 wellbeing scale), it is defined in varied ways and often with numerous distinct component outcomes that involve different measures including experienced hedonic factors (such as positive affect), evaluative hedonic factors (e.g., life satisfaction), and evaluative eudaimonic factors (such as purpose, meaning, mastery and coherence) (35). Given the breadth of this concept, common measures for wellbeing will be needed to enable evidence synthesis. In their review of SP outcomes and measures, Ashe et al. (30) identified six commonly-used wellbeing measures, with two—the Warwick-Edinburgh Mental Well-being Scale and the Short Warwick-Edinburgh Mental Well-being Scale—used most commonly. However, these are unidimensional measures, and it will be important to be able to further identify how SP programs differentially affect more specific aspects of wellbeing.

There will also be a need for studies of SP that gather new data, as opposed to reviewing patient records, as some of the outcomes in this KCO are not routinely collected in administrative data. Additionally, SP can have complex effects on some of the identified key outcomes, such as PCP visits. For example, while decreases in PCP visits can produce cost savings, increases can also be beneficial because people are acting on health symptoms earlier, rather than needing more expensive tertiary care.

As SP is intended to help reduce health inequities—including disparities in access to health-promoting resources that persist especially among un- or under-insured, low income, marginalized, Disabled, and Black and Brown people and communities—measurement of its impacts on health equity is critical. In this regard, and through the dialogue included in the Delphi Method process, the study identified a need for formative research to identify *what* outcomes related to equity can be addressed through the approach. The dialogue highlighted the importance of cultural equity and looking at the differences in service users’ values across diverse cultural backgrounds, inclusive of outcomes unique to individuals and to communities and groups.

### 4.3 Strengths and limitations

This study took a rigorous approach, akin to the development of a core outcomes set, to identifying key common outcomes. The Delphi Method processes were able to garner the input of both recognized leaders and experts in SP from around the world and a range of active stakeholders in SP in the US. The study established global perspectives that carried more experience than could be garnered from the US as a basis for consideration of the US context. The study was also strengthened by the combination of individual perspectives that the surveys invited, along with important input generated from the dialogic and consensus processes of the group discussions. The study was limited by the absence of SP service user perspectives. The group discussion yielded recognition of the importance of input from service users in determining what outcomes should be studied, and future COS work should include ample direct input from service users. This study was also limited by attrition in the final surveying round and lack of analysis of the measures used in SP outcomes research. Fortunately, the recent review conducted by Ashe et al. (30) offers an analysis of measures used in SP research. Additionally, reporting guidelines are needed for SP research in the US and globally.

## 5 Conclusion

This study identified outcomes that have been commonly studied in SP research across 13 countries and then used a two-phase Delphi Method process to develop a set of key common outcomes as a recommendation for focusing SP research in the US. The study identified wellbeing, social connection, and coping and resilience as the three top priority patient outcomes (among 10), and identified health equity, return on investment, and health system cost savings as the top three priority (among 6) health system outcomes. More consistent study of these key common outcomes has the potential to enable evidence synthesis of SP research and, in turn, drive evidence-based practice, investment, and policy in the US. Further research is needed to create a Core Outcomes Set for Social Prescribing in the US, along with reporting guidelines and common measurement sets.

## Data Availability

The raw data supporting the conclusions of this article will be made available by the authors, without undue reservation.
