# Supplementary material for: Identifying key outcomes for social prescribing research in the US: a Delphi method study
Source: Front Public Health. 2026 Aug 20;14:1761684. doi: 10.3389/fpubh.2026.1761684 (PMC13538844; doi:10.3389/fpubh.2026.1761684)
Supplement: Supplementary file 1 [file Table_1.DOCX]

Round #1 Survey #1

1. Please rank in priority order the importance of each of the 13 outcome categories. Drag and drop your choices into the box on the right.

| ______ Mental Health (patient) | Drag and drop your selections in order here. The box will expand as you add your choices. |
| --- | --- |
| ______ Lifestyle & behavior (patient) |  |
| ______ Patient/service user experience (patient) |  |
| ______ Relationships & social connection (patient) |  |
| ______ Physical health (patient) |  |
| ______ Community engagement & belonging (patient) |  |
| ______ Wellbeing (patient) |  |
| ______ Social determinants of health (patient) |  |
| ______ Healthcare and service utilization (system) |  |
| ______ Financial/economic (system) |  |
| ______ Workforce (system) |  |
| ______ Medication use/prescribing (system) |  |
| ______ General system outcomes (system) |  |

| Page Break |  |
| --- | --- |

2. Please identify your “top 10” outcomes from across the subcategories of patient and system outcomes. These are outcomes you think are important for study of social prescribing programs in the US, and should be ranked. Drag and drop your choices into the box on the right.

| ______ Coping and resilience (patient) | Drag and drop your selections here. The box will expand as you add your choices. |
| --- | --- |
| ______ Feelings and mood (patient) |  |
| ______ Symptoms (patient) |  |
| ______ Confidence, self-awareness, and identity (patient) |  |
| ______ Outlook (patient) |  |
| ______ Mental health-related quality of life (patient) |  |
| ______ Anxiety (patient) |  |
| ______ Depression (patient) |  |
| ______ Loneliness (patient) |  |
| ______ Condition management and health self-efficacy (patient) |  |
| ______ Daily behaviors (patient) |  |
| ______ Self-development (patient) |  |
| ______ Arts engagement (patient) |  |
| ______ Skills development (patient) |  |
| ______ Lifestyle & behavior (patient) |  |
| ______ Employment (patient) |  |
| ______ Substance use (patient) |  |
| ______ Goals (patient) |  |
| ______ Values and beliefs (patient) |  |
| ______ Dimensions of experience (patient) |  |
| ______ Satisfaction (patient) |  |
| ______ Access (patient) |  |
| ______ Participation (patient) |  |
| ______ Costs (patient) |  |
| ______ Social connection (patient) |  |
| ______ Social support (patient) |  |
| ______ Social engagement (patient) |  |
| ______ Relationships (patient) |  |
| ______ Physical activity (patient) |  |
| ______ Weight and BMI (patient) |  |
| ______ Diagnoses (patient) |  |
| ______ Physical wellness and abilities (patient) |  |
| ______ Belonging (patient) |  |
| ______ Community engagement (patient) |  |
| ______ Sense of community (patient) |  |
| ______ Community/group identification (patient) |  |
| ______ Connection (patient) |  |
| ______ Social isolation (patient) |  |
| ______ Physical wellbeing (patient) |  |
| ______ Social wellbeing (patient) |  |
| ______ Mental/emotional wellbeing (patient) |  |
| ______ Personal wellbeing (patient) |  |
| ______ Psychological wellbeing (patient) |  |
| ______ Quality of life (patient) |  |
| ______ Overall wellbeing (patient) |  |
| ______ Housing (patient) |  |
| ______ Employment (patient) |  |
| ______ Welfare/social support services (patient) |  |
| ______ Mental health and social care (system) |  |
| ______ Primary care/general practitioner (GP) visits (system) |  |
| ______ Healthcare usage (system) |  |
| ______ Emergency visits (system) |  |
| ______ Secondary care (system) |  |
| ______ Referrals (system) |  |
| ______ Costs (system) |  |
| ______ Cost savings (system) |  |
| ______ Return on investment (system) |  |
| ______ Workforce experiences and perceptions (system) |  |
| ______ Workload (system) |  |
| ______ Workforce outcomes (system) |  |
| ______ Workforce wellbeing (system) |  |
| ______ Prescriptions for medications (system) |  |
| ______ Medication use/compliance (system) |  |
| ______ Partnerships (system) |  |
| ______ Service integration (system) |  |
| ______ Health equity (system) |  |
| ______ Care options/resources (system) |  |

Round #1 Survey #2

1. Please rank in priority order the importance of each of the 13 outcome categories. Drag and drop your choices into the box on the right.

| ______ Mental Health (patient) | Drag and drop your selections in order here. The box will expand as you add your choices. |
| --- | --- |
| ______ Lifestyle & behavior (patient) |  |
| ______ Patient/service user experience (patient) |  |
| ______ Relationships & social connection (patient) |  |
| ______ Physical health (patient) |  |
| ______ Community engagement & belonging (patient) |  |
| ______ Wellbeing (patient) |  |
| ______ Social determinants of health (patient) |  |
| ______ Healthcare and service utilization (system) |  |
| ______ Financial/economic (system) |  |
| ______ Workforce (system) |  |
| ______ Medication use/prescribing (system) |  |
| ______ General system outcomes (system) |  |

2. Please identify your “top 10” outcomes from across the subcategories of patient and system outcomes. These are outcomes you think are important for study of social prescribing programs in the US, and should be ranked. Drag and drop your choices into the box on the right.

| ______ Coping and resilience (patient) | Drag and drop your selections here. The box will expand as you add your choices. |
| --- | --- |
| ______ Symptoms (patient) |  |
| ______ Confidence, self-awareness, and identity (patient) |  |
| ______ Outlook (patient) |  |
| ______ Mental health-related quality of life (patient) |  |
| ______ Anxiety (patient) |  |
| ______ Depression (patient) |  |
| ______ Loneliness (patient) |  |
| ______ Condition management and health self-efficacy (patient) |  |
| ______ Daily behaviors (patient) |  |
| ______ Arts engagement (patient) |  |
| ______ Skills development (patient) |  |
| ______ Lifestyle & behavior (patient) |  |
| ______ Employment (patient) |  |
| ______ Substance use (patient) |  |
| ______ Goals (patient) |  |
| ______ Dimensions of experience (patient) |  |
| ______ Satisfaction (patient) |  |
| ______ Access (patient) |  |
| ______ Participation (patient) |  |
| ______ Costs (patient) |  |
| ______ Social connection (patient) |  |
| ______ Physical activity (patient) |  |
| ______ Diagnoses (patient) |  |
| ______ Physical wellness and abilities (patient) |  |
| ______ Belonging (patient) |  |
| ______ Community engagement (patient) |  |
| ______ Sense of community (patient) |  |
| ______ Connection (patient) |  |
| ______ Social isolation (patient) |  |
| ______ Physical wellbeing (patient) |  |
| ______ Social wellbeing (patient) |  |
| ______ Mental/emotional wellbeing (patient) |  |
| ______ Psychological wellbeing (patient) |  |
| ______ Quality of life (patient) |  |
| ______ Overall wellbeing (patient) |  |
| ______ Housing (patient) |  |
| ______ Welfare/social support services (patient) |  |
| ______ Mental health and social care (system) |  |
| ______ Healthcare usage (system) |  |
| ______ Referrals (system) |  |
| ______ Costs (system) |  |
| ______ Cost savings (system) |  |
| ______ Return on investment (system) |  |
| ______ Workforce experiences and perceptions (system) |  |
| ______ Workforce wellbeing (system) |  |
| ______ Prescriptions for medications (system) |  |
| ______ Medication use/compliance (system) |  |
| ______ Partnerships (system) |  |
| ______ Service integration (system) |  |
| ______ Health equity (system) |  |
| ______ Care options/resources (system) |  |

Round #1 Demographics & Information Survey

Thank you for your interest in the Key Common Outcomes for Social Prescribing focus group! Please complete the following fields.

Name

________________________________________________________________

Email address

________________________________________________________________

Organizational Affiliation

________________________________________________________________

Primary discipline:

- Healthcare
- Public health
- Arts and culture
- Other __________________________________________________

Primary role related to social prescribing (SP) :

- SP manager
- SP program provider
- SP researcher
- Other __________________________________________________

| Page Break |  |
| --- | --- |

How would you primarily describe yourself? If you identify as two or more races/ethnicities, you will be given an opportunity to make multiple selections in the next question.

- Native Hawaiian or Pacific Islander
- Native American or Native Alaskan
- Asian or Asian American
- Black or African American
- White or Caucasian
- Latino/a/x or Hispanic origin, such as Mexican, Puerto Rican, or Cuban
- Other
- I identify as two or more races/ethnicities
- Prefer not to answer

Display This Question:

If How would you primarily describe yourself? If you identify as two or more races/ethnicities, you... = I identify as two or more races/ethnicities

Please select all of the races/ethnicities you would use to describe yourself.

- Native Hawaiian or Pacific Islander
- Native American or Native Alaskan
- Asian or Asian American
- Black or African American
- White or Caucasian
- Latino/a/x or Hispanic origin, such as Mexican, Puerto Rican, or Cuban
- Other

What is your gender identity?

- Gender non-conforming
- Woman (cisgender or transgender)
- Non-binary
- Man (cisgender or transgender)
- Identity not listed
- Prefer not to answer

What is your age?

________________________________________________________________

What is the highest level of school you have completed or the highest degree you have received?

- Less than a high school degree
- High school graduate (high school diploma or equivalent including GED)
- College degree
- Graduate or Professional degree

Relative to others in your community, how would you describe your income level?

- Low income
- Middle income
- High income

Round #2 Survey #3

Key Common Outcomes for Social Prescribing Survey
Protocol #: ET00022727
Information and Consent

This survey is part of a research study being conducted by the EpiArts Lab at the University of Florida, a National Endowment for the Arts research lab in partnership with researchers at University College London.

This survey is intended to identify a set of key common outcomes that should be studied in relation to social prescribing and arts on prescription programs in the United States (US). It is intended to encourage studies of priority outcomes in the US as a means for strengthening the evidence base and enabling evidence synthesis that can guide evidence-based practice, policy and investment in social prescribing and Arts on Prescription in the US.

The survey will ask you 11 question in two parts – part one will includes five professional and demographic questions, and part two includes six questions related to priority patient and system outcomes for social prescribing and arts on prescription research. This survey should take you approximately 6-10 minutes to complete.

Your participation in this research is confidential. Information collected about you will be stored in computers with security passwords. Only the study team will have the right to review these research records, and they will protect the secrecy (confidentiality) of these records.

There are no risks to participating in the study. You have the right to stop the survey at any point.

A few weeks after you complete this survey, we'll share the results with you and invite you to complete the survey again, taking those results and others' perspectives into consideration. Your participation in this second round is completely optional. 

If you have any questions, please contact our Principal Investigator, Dr. Jill Sonke, at jsonke@ufl.edu. You may also contact the UF IRB at (352)-392-0433.

By selecting yes, you are confirming that you are 18 years old or older and consent to participate in this survey.

- Yes
- No

1. Primary discipline:

- Healthcare
- Public health
- Arts and culture
- Other __________________________________________________

2. Primary role(s) related to social prescribing (SP) :

- SP manager or administrator
- SP program/service provider
- SP researcher
- SP link worker, community health worker or other program liaison (someone who connects people to services)
- Other __________________________________________________

How long have you been associated with social prescribing?

________________________________________________________________

3. How would you primarily describe yourself? Select all that apply.

- Native Hawaiian or Pacific Islander
- Native American or Native Alaskan
- Asian or Asian American
- Black or African American
- White or Caucasian
- Latino/a/x or Hispanic origin, such as Mexican, Puerto Rican, or Cuban
- Other, please describe __________________________________________________
- Prefer not to answer

4. What is/are your gender identity(ies)?

- Gender non-conforming
- Woman (cisgender or transgender)
- Non-binary
- Man (cisgender or transgender)
- Identity not listed; please provide if you would like: __________________________________________________
- Prefer not to answer

End of Block: Block 1

Start of Block: Block 2

Part Two:

Introduction: Social prescribing is “a means for trusted individuals in clinical and community settings to identify that a person has non-medical, health-related social needs and to subsequently connect them to non-clinical supports and services within the community by co-producing a social prescription—a non-medical prescription, to improve health and well-being and to strengthen community connections” (Muhl et al., 2023).

Arts on Prescription programs generally hold the same goals and focus programming on arts, culture and nature (Golden et al., 2023). Please consider this definition and how research on these programs can help these goals to be achieved as you complete this survey.

Based on a mapping review of the social prescribing outcomes literature from 13 countries, which you can access here, and a Delphi Method study undertaken with global social prescribing experts, the outcomes presented in this survey have been identified as both the most commonly studied and as priority for study from a global perspective.

The outcomes are organized into two categories - patient outcomes and system outcomes - and are presented for your consideration below. The next six questions will ask you to rank the priority of outcomes in each category and invite you to provide comments.

| Page Break |  |
| --- | --- |

7. Patient Outcomes: From your perspective, please rank the following patient outcomes in priority order, from most important to least important.

______ Wellbeing

______ Condition management

______ Health self-efficacy

______ Social connection

______ Coping and resilience

______ Belonging

______ Loneliness

______ Physical activity

______ Social determinants of health

______ User experience

8. Are there any outcomes missing? If so, which one(s) and why are they important?

________________________________________________________________

________________________________________________________________

________________________________________________________________

________________________________________________________________

________________________________________________________________

9. Please comment on your choices. Feel free to advocate for particularly important outcomes or your rationale for your choices.

________________________________________________________________

________________________________________________________________

________________________________________________________________

________________________________________________________________

________________________________________________________________

End of Block: Block 2

Start of Block: Block 4

10. System Outcomes: From your perspective, please rank the following system outcomes in priority order, from most important to least important.

______ Return on Investment

______ Health equity

______ Health system cost savings

______ System utilization

______ GP visits

______ Workforce outcomes

11. Are there any outcomes missing? If so, which one(s) and why are they important?

________________________________________________________________

________________________________________________________________

________________________________________________________________

________________________________________________________________

________________________________________________________________

12. Please comment on your choices. Feel free to advocate for particularly important outcomes or your rationale for your choices.

________________________________________________________________

________________________________________________________________

________________________________________________________________

________________________________________________________________

________________________________________________________________

Round #2 Survey #4

Key Common Outcomes for Social Prescribing Survey
Protocol #: ET00022727
Information and Consent

This survey is part of a research study being conducted by the EpiArts Lab at the University of Florida, a National Endowment for the Arts research lab in partnership with researchers at University College London.

This survey is intended to identify a set of key common outcomes that should be studied in relation to social prescribing and arts on prescription programs in the United States (US). It is intended to encourage studies of priority outcomes in the US as a means for strengthening the evidence base and enabling evidence synthesis that can guide evidence-based practice, policy and investment in social prescribing and Arts on Prescription in the US.

This survey serves as the second survey of a Delphi testing process. In the first survey, you ranked priority outcomes related to social prescribing. In this survey, you will be asked to review the results from the first round to inform a new set of rankings. You will also be asked 5 professional and demographic questions.  This survey should take you approximately 6-10 minutes to complete.

Your participation in this research is confidential. Information collected about you will be stored in computers with security passwords. Only the study team will have the right to review these research records, and they will protect the secrecy (confidentiality) of these records.

There are no risks to participating in the study. You have the right to stop the survey at any point.

If you have any questions, please contact our Principal Investigator, Dr. Jill Sonke, at jsonke@ufl.edu. You may also contact the UF IRB at (352)-392-0433.

By selecting yes, you are confirming that you are 18 years old or older and consent to participate in this survey.

- Yes
- No

End of Block: Default Question Block

Start of Block: Block 1

1. Primary discipline:

- Healthcare (1)
- Public health (2)
- Arts and culture (3)
- Other (4) __________________________________________________

2. Primary role(s) related to social prescribing (SP) :

- SP manager or administrator (1)
- SP program/service provider (2)
- SP researcher (3)
- SP link worker, community health worker or other program liaison (someone who connects people to services) (5)
- Other (4) __________________________________________________

How long have you been associated with social prescribing?

________________________________________________________________

3. How would you primarily describe yourself? Select all that apply.

- Native Hawaiian or Pacific Islander (1)
- Native American or Native Alaskan (2)
- Asian or Asian American (3)
- Black or African American (4)
- White or Caucasian (5)
- Latino/a/x or Hispanic origin, such as Mexican, Puerto Rican, or Cuban (6)
- Other, please describe (7) __________________________________________________
- Prefer not to answer (9)

4. What is/are your gender identity(ies)?

- Gender non-conforming (1)
- Woman (cisgender or transgender) (2)
- Non-binary (3)
- Man (cisgender or transgender) (4)
- Identity not listed; please provide if you would like: (5) __________________________________________________
- Prefer not to answer (6)

End of Block: Block 1

Start of Block: Block 2

Part Two:

As a reminder, social prescribing is “a means for trusted individuals in clinical and community settings to identify that a person has non-medical, health-related social needs and to subsequently connect them to non-clinical supports and services within the community by co-producing a social prescription—a non-medical prescription, to improve health and well-being and to strengthen community connections” (Muhl et al., 2023). Arts on Prescription programs generally hold the same goals and focus programming on arts, culture and nature (Golden et al., 2023).

In the first Delphi Method survey round, the options we presented were based on a mapping review of the social prescribing outcomes literature from 13 countries, which you can access [here](https://www.frontiersin.org/articles/10.3389/fmed.2023.1266429/full), and were organized into two categories - patient outcomes and system outcomes.

21 experts on social prescribing in the US completed the first survey. We ask you below to consider the results and to offer any changes you think should be made.

| Page Break |  |
| --- | --- |

For the first survey round, we asked you to rank the priority of patient outcomes. Here are the results from that survey (in ranked order):

#1. Wellbeing
#2. Social connection
#3. Coping and resilience
#4. Social determinants of health
#5. Health self-efficacy
#6. Belonging 
#6. Loneliness
#7. Condition management 
#8. User experience
#9. Physical activity

7. Please make any changes you would like to this ranked list of patient outcomes below by dragging and dropping them.

______ Wellbeing (1)

______ Social connection (4)

______ Coping and resilience (5)

______ Social determinants of health (9)

______ Health self-efficacy (3)

______ Belonging (6)

______ Loneliness (7)

______ Condition management (2)

______ User experience (10)

______ Physical activity (8)

8. Are there any outcomes missing? If so, which one(s) and why are they important?

________________________________________________________________

________________________________________________________________

________________________________________________________________

________________________________________________________________

________________________________________________________________

9. Please comment on your choices. Feel free to advocate for particularly important outcomes or your rationale for your choices.

________________________________________________________________

________________________________________________________________

________________________________________________________________

________________________________________________________________

________________________________________________________________

End of Block: Block 2

Start of Block: Block 4

For the first survey round, we asked you to rank the priority of system outcomes. Here are the results from that survey ( in ranked order):

#1. Return on Investment

#2. Health Equity

#3. Health system and cost savings

#4. System utilization

#5. GP visits

#6. Workforce outcomes

10. Please make any changes you would like to this ranked list or system outcomes below.

______ Return on Investment (1)

______ Health equity (2)

______ Health system cost savings (3)

______ System utilization (4)

______ GP visits (5)

______ Workforce outcomes (6)

11. Are there any outcomes missing? If so, which one(s) and why are they important?

________________________________________________________________

________________________________________________________________

________________________________________________________________

________________________________________________________________

________________________________________________________________

12. Please comment on your choices. Feel free to advocate for particularly important outcomes or your rationale for your choices.

________________________________________________________________

________________________________________________________________

________________________________________________________________

________________________________________________________________

________________________________________________________________

End of Block: Block 4
